# Recruitment in Appalachian, Rural and Older Adult Populations in an Artificial Intelligence World: Study Using Human-Mediated Follow-Up

**DOI:** 10.2196/38189

**Published:** 2024-08-22

**Authors:** Tabitha Milliken, Donielle Beiler, Samantha Hoffman, Ashlee Olenginski, Vanessa Troiani

**Affiliations:** 1 Research Institute Geisinger Danville, PA United States; 2 Philadelphia College of Osteopathic Medicine Philadelphia, PA United States

**Keywords:** telecommunication, enrollment rate, Northern Appalachia, web-based, aging, recruitment, rural

## Abstract

**Background:**

Participant recruitment in rural and hard-to-reach (HTR) populations can present unique challenges. These challenges are further exacerbated by the need for low-cost recruiting, which often leads to use of web-based recruitment methods (eg, email, social media). Despite these challenges, recruitment strategy statistics that support effective enrollment strategies for underserved and HTR populations are underreported. This study highlights how a recruitment strategy that uses email in combination with follow-up, mostly phone calls and email reminders, produced a higher-than-expected enrollment rate that includes a diversity of participants from rural, Appalachian populations in older age brackets and reports recruitment and demographic statistics within a subset of HTR populations.

**Objective:**

This study aims to provide evidence that a recruitment strategy that uses a combination of email, telephonic, and follow-up recruitment strategies increases recruitment rates in various HTR populations, specifically in rural, older, and Appalachian populations.

**Methods:**

We evaluated the overall enrollment rate of 1 recruitment arm of a larger study that aims to understand the relationship between genetics and substance use disorders. We evaluated the enrolled population’s characteristics to determine recruitment success of a combined email and follow-up recruitment strategy, and the enrollment rate of HTR populations. These characteristics included (1) enrollment rate before versus after follow-up; (2) zip code and county of enrollee to determine rural or urban and Appalachian status; (3) age to verify recruitment in all eligible age brackets; and (4) sex distribution among age brackets and rural or urban status.

**Results:**

The email and follow-up arm of the study had a 17.4% enrollment rate. Of the enrolled participants, 76.3% (4602/6030) lived in rural counties and 23.7% (1428/6030) lived in urban counties in Pennsylvania. In addition, of patients enrolled, 98.7% (5956/6030) were from Appalachian counties and 1.3% (76/6030) were from non-Appalachian counties. Patients from rural Appalachia made up 76.2% (4603/6030) of the total rural population. Enrolled patients represented all eligible age brackets from ages 20 to 75 years, with the 60-70 years age bracket having the most enrollees. Females made up 72.5% (4371/6030) of the enrolled population and males made up 27.5% (1659/6030) of the population.

**Conclusions:**

Results indicate that a web-based recruitment method with participant follow-up, such as a phone call and email follow-up, increases enrollment numbers more than web-based methods alone for rural, Appalachian, and older populations. Adding a humanizing component, such as a live person phone call, may be a key element needed to establish trust and encourage patients from underserved and rural areas to enroll in studies via web-based recruitment methods. Supporting statistics on this recruitment strategy should help researchers identify whether this strategy may be useful in future studies and HTR populations.

## Introduction

The success of human subject research is reliant on effective recruitment strategies. Without successful recruitment, human subject research cannot be accomplished, yet the recruitment process itself can present an obstacle for researchers [1-3]. Common barriers to patient recruitment include mistrust of research, perceived harms, cost, patient location (eg, rural area), transportation, medical illiteracy, lack of research opportunities, time, and family objection [4-7]. Given the difficulties associated with recruitment, one might anticipate an increase in research centered on successful recruitment strategies and retention statistics in specific target populations; especially hard-to-reach (HTR) populations. In research, HTR populations describe subgroups that are difficult to contact due to economical, geographical, physical, or social situations [8].

Recruitment methods commonly include flyers and other printed advertisements (eg, newspapers), but since web-based access has become commonplace, many strategies involve some form of web-based recruitment. For example, emails may be sent that include information about a study that would have typically required posted mail in the past, and posts on social media platforms (eg, Facebook) are now more widely used to reach populations that a researcher is interested in recruiting. While many recruitment strategies are used in human subjects research, data about specific types of recruitment strategies (eg, social media, email, telephone, postal, patient portal, combination) with supporting recruitment statistics and evidence of efficacy are often inconsistent, vary based on target populations, and often limited in HTR populations [1]. Many of the existing review articles regarding enrollment rates cite the need for additional literature that supports efficient recruitment methods in specific populations, especially HTR populations [2,9,10]. Web-based or digital recruitment has become popular, as roughly 6%-50% of studies use web-based methods [11], but web-based methods often fail to reach the diversity of patient populations [12]. One study that aimed to provide support to effective and equitable recruitment methods found that unsolicited recruitment emails had a low 6.1% enrollment rate and telephone calls had 37.8% enrollment [9]. Another study found that older populations and marginalized groups were often underrepresented in digital approaches, thus necessitating further research to understand recruitment barriers and successful recruitment strategies [2,9]. Using a successful recruitment strategy is even more important when targeting marginalized or HTR populations [2,8,9]. HTR populations face additional barriers that can make recruitment arduous, while at the same time, are the very populations whose participation in research is critical to fill gaps in knowledge [4]. Thus, identifying efficient recruitment strategies that result in successful enrollment of HTR and other targeted populations is important.

One specific HTR population includes individuals residing in Appalachia. Appalachia is a 205,000–square mile region that follows the Appalachian Mountains from northern Mississippi to southern New York and is home to more than 25 million people [13,14]. Appalachia is a federally designated region that is characterized by environmental and socioeconomic factors that include low education, low income, limited health literacy, lack of access to health care, and challenging rural geography [7,14]. This region has been impacted by geographical isolation and natural resource exploitation and, as such, the people of Appalachia often have an “insider versus outsider” mentality and are mistrustful of outsiders and the medical community [1,13,14]. This mentality extends into many rural communities and the mistrust of the medical community is common among older adults [5,13]. Patients enrolled into studies from rural areas, such as many parts of Appalachia that are extremely rural and geographically isolated, are often less educated, from lower socioeconomic backgrounds, and lack the medical literacy necessary to clearly understand research [1,6,7,13]. There are numerous infrastructure barriers, including transportation availability, distance, and roads, as well as web-based access, that can also hinder recruitment [1,13]. Older adults, aged 60 years and older, are a common HTR subgroup and share many of the same recruitment barriers as rural populations. Older adults can be more difficult to recruit through digital or web-based methods due to reduced web-based access, attitudes toward technology, and age-related medical issues such as hearing and vision loss [2,15]. As a result, recruitment and effective recruitment strategies of Appalachian populations, as well as rural and older adult populations, can be difficult and are limited in the literature [13].

There are numerous studies about the health disparities and recruitment barriers in the Appalachian population [1,6,16-18]; however, there are limited literature and data specifically about recruitment strategies that demonstrate efficient and successful recruitment into studies that target Appalachian, rural-dwelling, and older adult populations. Studies that target HRT populations often state the need for cultural competency and trustworthiness, which can be accomplished by human-mediated outreach (physical or digital) [2,6,15,16]. Existing studies that did address web-based recruitment typically used social media and email but lacked human-mediated follow-up [9-11,19]. Information about the demographics of enrollees was not always detailed and depended heavily on what the researchers were investigating. Studies that did report information and enrollment rate statistics were widely inconsistent and varied in target population, sample size, and media type [2,11,12]. A recent review by Frampton et al [2] summarized existing papers that used digital tools for the recruitment and retention of participants in randomized controlled trials. Of the 104 articles in the review, only 16 focused on minority or underserved populations [20-35] with 14 deemed HTR populations [22-25,27-29,33,35-40] by the authors of this paper. Only 3 articles included populations in the Appalachian region (Scranton, Pennsylvania [PA], and Pittsburgh, PA) [41-43], but Appalachian populations were not the primary target in any of these studies. The review had 9 studies that focused on rural areas and counties [41,44-51]; however, only 2 of the studies conducted in rural areas were located the United States [41,50], with the other studies occurring in rural and remote parts of the United Kingdom and Australia. Ten of the studies reviewed used human-mediated calls for recruitment, with recruitment rates varying from 0% to 88% in these studies [40,52-59]. It must be noted though, of the 10 studies that used human-mediated calling, human-mediated calls were not the only methods of recruitment. Social media and web advertisements were used in 7 of the 10 studies [40,53-55,58-60]. Studies that used social media had higher overall enrollment rates but enrollment rates using telephone alone ranged between 0% and 17% [47-51,53]. Overall, authors of this review concluded that there is limited literature about efficacy of recruitment strategies, and further research is needed to determine which digital or nondigital tools and combinations would be best suited for specific populations [2]. While HTR populations are being included in research more readily, the primary focus is more directed at underrepresented racial, ethnic, sex, and LGBTQIA+ (lesbian, gay, bisexual, transgender, queer, intersex, and asexual) groups, with less focus on other marginalized groups such Appalachian residents, rural-dwelling residents, and older adults [19-29,40,61-63].

This study's main goal is to add knowledge to the field regarding a recruitment strategy that enrolled participants from Appalachian, rural-dwelling, and older adult populations in a rural PA health care system. The secondary goal is to determine whether adding participant follow-up yielded higher enrollment numbers than without participant follow-up. The novelty of this study is the outreach of this recruitment strategy in Appalachian, rural-dwelling, and older adult populations and the study focus on substance abuse and genetics [64]. In a recent review article [2], only 2 cited studies focused on recruitment of Appalachian populations [41,42] and none on genetics and substance abuse. The Frampton’s review had 13 studies that focused on substance abuse (primarily alcohol and tobacco) [44,45,65-73], and only one of the studies was recruiting from biobank samples [56]. The target enrollment number for the overall study is 20,000 people (enrollment ongoing), and this email recruitment is one of several recruitment arms used in the study [20]. Email recruitment often reaches larger amounts of people than mailings and face-to-face recruitment [2,9,10,13], and follow-up has been shown to increase enrollment in a study that did postal mailings [74]. Thus, the email and follow-up enrollment strategies described here may be helpful to adapt in studies of similar populations of older adults and rural Appalachian residents, as well as other HTR populations.

## Methods

### Participants

This study was conducted at Geisinger, an integrated health system based in central, south-central, and northeastern PA, that serves about 3.8 million patients, including older adults in rural Appalachia. PA has 67 counties, with 48 designated as rural, 19 designated as urban, and 52 designated as Appalachian. To identify the available recruitment population for this study, any patient with a Geisinger health record was screened for eligibility. Patients were still eligible if they had moved outside of the service area, which included Appalachian and rural counties outside the specified service area. Recruitment data are based on the county of current residence. In 2020, approximately 3.4 million people or 26% of the state’s population lived in rural counties and 64% of rural municipalities in PA had fewer than 2500 residents [75,76]. In addition, PA has 2.7 million (21.3%) people older than 60 years and 1.4 million (11.1%) people older than 70 years as of 2012 [76]. Data used in this manuscript were collected from July 2018 to July 2021 and recruitment is ongoing for an internal review board–approved study, the design of which has been published previously [64]. A US $10 electronic gift card was offered to participants who completed the survey as compensation for their time.

For the analyses described here, we focus on 1 specific recruitment arm of the study, in which recruitment letters were sent to potential participants who met eligibility criteria via a secure email, patient portal message, and paper copy. A waiver of consent was obtained for screening and recruitment, and names, addresses, phone numbers, email addresses, and medical record numbers of patients who met the inclusion criteria were obtained through a Geisinger data broker. The inclusion criteria consist of patients in the Geisinger Health System between the ages of 18 and 75 years and have received at least 2 opioid prescriptions over their lifetime. Participants must be of European ancestry, be English speaking, be enrolled in Geisinger’s biobank [77], and with no history of metastatic cancer.

### Recruitment

Email letter recruitment batches of 1-499 emails were sent from a secure REDCap (Research Electronic Data Capture) platform. REDCap is a secure, web-based software platform designed to support data capture for research studies and is approved to house-protected health information, including patient contact information, which can be used to send automated email invitations to participate in research [78,79]. Following the initial email, a reminder email was sent after 1 week. Any patient who did not complete or did not decline the survey after the automated follow-up email received a follow-up phone call. If a patient answered the telephone and was interested in completing the survey, the recruitment email with the survey link or a secure patient portal message containing the survey link was resent. Patients who expressed interest but did not have access to a computer or the internet were offered the option to do the survey over the phone or receive a paper packet. Paper packets included hard copies of the consent, survey, and a prepaid envelope for participants to return the survey. Patients who were not interested were thanked for their time, recorded as a “decline,” and not recontacted. If a patient did not answer, a brief voicemail message containing study details and contact information was left. If voicemail was not an option, 1 additional reminder email was sent as the third contact attempt. See Textbox 1 for an overview of the recruitment process.

Patient contacts across modalities (email, phone, patient portal, and paper packets) were limited to 3 contacts. Patients who received more than 3 contacts were patients who partially completed the survey, requested a link resend, or requested a paper packet and never returned it. Partial completion patients received an additional reminder email with an access code to the survey and an additional follow-up phone call. Patients who requested a paper packet received a follow-up phone call if they did not return the packet within several months or if they returned an incomplete survey and consent form. If a link resend was requested, these patients received 1 additional follow-up email reminder. Information about the email and follow-up call responses were recorded in REDCap, including patient status and call status (message, enrolled, declined, link resend, etc), date of consent, and any relevant notes about the patient or call.

**Eligibility requirements and step-by-step recruitment process for recruitment into the study via email and follow-up.**

- **Eligible patients are sent a recruitment letter via REDCap (Research Electronic Data Capture).**
  Eligibility requirements: 18-75 years, European descent, >1 opioid prescription, current enrollment in Geisinger biobank, read and write fluent English, no metastatic cancer.
- Recruitment letter contains a link to complete a 20- to 30-minute survey.
- Reminder email is sent 1-week later.
- **Research assistant (RA) calls patients who have not completed the survey 1 day after reminder email. Patients receive only 1 follow-up call.**
  If no answer, RA leaves a ~30-second voicemail that contains the study name and contact information.
  If a patient answers, the RA explains the study and asks whether the patient is interested.
- **Interested patients are sent another email.**
  Patients who start the survey and stop part way are also sent an additional reminder with an access code so that they may finish.
  Patients who are not interested are marked as a “decline” in our database.
- Patients who complete the survey are compensated with a US $10 gift card

### Survey

Participants received an email link and were asked to complete surveys via the email link on a personal computer or other digital device, by phone interview, or by requesting a paper packet to be mailed. Questionnaires include the Brief Risk Questionnaire, Fagerstrom Test for Nicotine Dependence, Alcohol Use Disorder Identification Test, Generalized Anxiety Disorder 7-Item, Patient Health Questionnaire-9, and questions derived from the criteria for opioid use disorder diagnosis from the *DSM-5* (*Diagnostic and Statistical Manual of Mental Disorders* [Fifth Edition]), among others [64]. After the questionnaires are completed, there is no further study-related follow-up or patient contact.

### Enrollment Demographics

The overall enrollment rate and population characteristics were evaluated to determine recruitment success of a combined email and follow-up recruitment strategy and its outreach in Appalachian and older adult populations.

The characteristics evaluated included (1) enrollment rate before versus after research assistant (RA) follow-up; (2) zip code and county of enrollee to determine rural status; (3) age to verify recruitment in all eligible age brackets; and (4) sex distribution in rural versus urban counties and age brackets.

1. *Overall enrollment rate*: to determine the overall enrollment rate, the number of completed surveys was divided by the total number of patients contacted for enrollment.
2. *Recruitment strategy*: Success of the combined email and follow-up strategy was determined by separating enrollees into 2 groups: those completing the survey before follow-up and those completing the survey after follow-up. Once in these categories, a percentage of enrollment in each category was determined.
3. *Rural versus urban*: outreach into rural PA was calculated by cross-referencing zip codes of enrolled patients against a list of all PA zip codes and sorting patients into counties. Enrollees with zip codes in a rural county were classified as “rural” and enrollees with a zip code in an urban county were classified as “urban.” Rural and urban designations were based on population density being either below 291 persons per square mile (rural) or above 291 persons per square mile (urban) [75]. With this information, the number and percentage of patients enrolled in rural and urban PA counties were determined. PA counties were designated as Appalachian and non-Appalachian counties, based on the Appalachian Regional Commission designations [14].
4. *Age*: patients were separated into 6 different age brackets, based on age at date of consent. The distribution among the different age brackets allowed us to determine whether our recruitment strategy was reaching older adults.
5. *Sex distribution*: enrolled patients were divided by self-reported sex and then further divided by sex and age bracket, sex, age bracket, and rural versus urban distribution. These distributions allow for further examination of underserved populations in each age, sex, and geographic designation.

Enrollment rate before and after follow-up for each recruitment batch in a series of 74 batches was compared to determine whether participant follow-up yielded greater enrollment using a paired *t* test. Descriptive statistics, separated by age brackets, sex, and geographic region, were used to summarize data across the cohort.

### Ethical Considerations

Ethical approval for the overall study was obtained from the Geisinger internal review board (study number 2017-0190). All participants received a thorough explanation of the study consent form and were given the opportunity to ask questions prior to and during enrollment. All participants were asked to complete a digital consent form during enrollment. Participants are free to withdraw from the study at any time. Participants were reimbursed US $10 for their time and effort, which was paid for using funds from the National Institutes of Health (NIH) grant (R01DA044015) and from a grant from the Pennsylvania Department of Health. A certificate of confidentiality has been obtained from the NIH for this study. Identifiable data from the study will not be shared with participants’ doctors. All patient data are stored on a HIPAA (Health Insurance Portability and Accountability Act)-compliant portal. All study staff interacting with potential or enrolled participants received training on human subjects research [64].

## Results

From July 2018 to July 2021, a total of 6106 participants completed the survey via the email and follow-up recruitment arm of the overall study. Approximately 35,037 patients were contacted, resulting in a 17.4% (6106/35037) enrollment rate. Approximately 37.6% (2297/6106) of the participants enrolled before the follow-up and 62.4% (3809/6106) enrolled after follow-up by an RA, either by phone call or by additional email follow-up or correspondence. In addition, of the 3809 enrollees who had RA follow-up, 87% (3313/3809) had email and telephonic follow-up, 13% (497/3809) enrolled after 1 voicemail and no additional RA contact or follow-up, 3.7% (141/3809) enrolled via paper, and 2.6% (10/3809) participants enrolled over the phone.

The email and follow-up recruitment strategy enrolled patients in 64% (43/67) of PA counties and 66% (32/48) of rural PA counties. The study also enrolled 72 patients in 25 states outside of PA. Non-PA participants were excluded from this assessment, which resulted in the inclusion of 6030 patients for age, sex, rural versus urban, and Appalachian data. Of participants who enrolled from rural PA counties, 76.30% (4603/6030) enrolled from rural PA Appalachian counties. Only 1 patient living in a rural PA county did not live in a rural PA Appalachian county. Appalachia includes some urban areas and 22.47% (1355/5956) of the Appalachian population were from urban PA Appalachian counties. In total, 98.77% (5956/6030) of the enrolled population were from PA Appalachian counties and 1.26% (76/6030) were not. See Table 1 for the breakdown of the enrolled population as Appalachian or non-Appalachian, Table 2 for the demographic breakdown of the enrolled population, and Table S1 in Multimedia Appendix 1 for more fine-grained demographic breakdown of rural versus urban enrollees.

Female enrollment represented 72.49% (4371/6030) of the total inclusion population and male enrollment represented 27.5% (1659/6030) of the total inclusion population. The average age at enrollment was 54.9 (SD 13.3) years, with ages ranging from 20.2 to 75.9 years. The 60-70 years age bracket enrolled the most patients, including male and female participants. Of note, no patients between the ages of 18 and 19 years enrolled via the email and follow-up arm of the overall study. As a result, the 18-19 years age bracket was excluded. Sex was based on patient identification of binary sex, male or female, via the health record. See Table S1 in Multimedia Appendix 1 for a breakdown of age brackets by urban and rural designation.

**Table 1:** Characteristics of rural and urban Appalachian and non-Appalachian counties for all participants recruited via email and follow-up method.

	Appalachia, n (%)	Non-Appalachia, n (%)	Total, n (%)
Rural	4601 (76.3)	1 (0.02)	*4602 (76.3)^a^*
Urban	1355 (22.5)	73 (1.2)	*1428 (23.7)^a^*
Total	*5956 (98.7)^a^*	*74 (1.3)^a^*	*6030 (100)^a^*

^a^Values in italics indicate that significance testing was not performed on the data.

**Table 2:** Demographic characteristics of participants enrolled via email and follow-up recruitment by age and sex.

Age range (years)	Female, n (%)	% of total population	Male, n (%)	% of total population	Total, n (%)
20-30	191 (4.37)	3.17	23 (1.4)	0.38	214 (3.55)
30-40	718 (16.43)	11.91	132 (8)	2.19	850 (14.10)
40-50	837 (19.15)	13.88	219 (13.1)	3.63	1056 (17.51)
50-60	1039 (23.77)	17.23	379 (22.9)	6.29	1418 (23.52)
60-70	1077 (24.64)	17.86	552 (33.2)	9.15	1629 (27.01)
70-75	509 (11.64)	8.44	354 (21.4)	5.87	863 (14.31)
Total	4371 (100)	72.49	1659 (100)	27.51	6030 (100)

## Discussion

### Principal Results

The primary aim of this analysis was to determine whether an email and follow-up recruitment strategy is reaching HTR populations: namely, rural, Appalachian, and older adult populations. Our secondary aim is to determine whether the follow-up component of our recruitment strategy (live person calling and email correspondence) increased enrollment numbers in these populations. In addition to these aims, a novel component of this study is the in-depth reporting of the strategy used; while many review articles and publications mention the need for this type of study, few exist for this enrollment strategy and the enrolled populations. We report a recruitment strategy that reached the historically marginalized Appalachian population, as well as other rural-dwelling residents and older adults.

Most patients reached using the methods described here resided in PA Appalachian counties and other rural residences and were classified as older adults. Thus, this study indicates that previously reported mistrust that is common in Appalachian and rural populations [1,13,16] and the negative attitudes toward technology found in older adults [4,15] may be overcome using similar recruitment strategies. We also found that more participants enrolled after human-mediated follow-up than with just email alone. We expected the email and follow-up recruitment arm of the study to yield enrollment rates between 6% and 10%, based on conservative estimates from NIH and Institute of Medicine [80]. However, the email and follow-up recruitment strategy yielded a percentage of enrollment nearly double (17.4%) this expectation. There was a significant difference between mean enrollment before RA follow-up with a mean of 30.6 (SD 11.17) participants per batch and mean enrollment after RA follow-up of 50.77 (SD 19.31) participants per batch; conditions (t_74_=1.99; *P*<.001). With this information, we rejected the null hypothesis that follow-up does not significantly affect web-based recruitment enrollment. This led us to determine that follow-up to email recruitment, primarily in the form of live person telephonic calls and additional email correspondence, significantly increases participant enrollment in web-based recruiting studies compared with email recruitment alone. Our live person telephonic call and email follow-up strategy may be useful in reaching other rural and marginalized populations via web-based method. It may also be useful for situations where digital recruitment is a necessity, such as during the COVID-19 pandemic. Recruitment using this method continued throughout the entire COVID-19 pandemic and proved to be highly adaptable for recruitment from home [77].

Our study was not designed to understand the specific reasons for the success of these recruitment strategies in HTR populations. However, we speculate that the trust and connection offered by a live person phone call may contribute to the success of this recruitment strategy [3,16,61,81]. Of the participants who enrolled after follow-up, 87% received a phone call and additional email correspondence. We speculate that having a live person call via telephone may validate the legitimacy of the study to potential participants and establishes a point of contact and component of trust. Participants can be reassured that the study is legitimate, not a scam, and have confidence that any questions will be answered by a knowledgeable study team member in a timely manner. This humanizing component and the element of trust may be necessary for recruiting HTR populations via digital or web-based recruiting methods, especially in rural, Appalachian, and older adult populations [1,5,6,8,52,81]. Future qualitative studies should explore the specific reasons for the success of this strategy.

For older adults, rural-dwelling and Appalachian populations, general distrust of health care providers and researchers, fear of being taken advantage of, medical/health/research illiteracy, poverty, transportation, chronic health conditions and technology constraints (cell phones or internet) are common barriers to study enrollment [5,16,35,82]. By following up with a patient via phone and email correspondence instead of a singular email, participants can establish study legitimacy, ask questions to a real person, and have the study explained verbally in understandable language and request accommodations, such as an over the phone or paper survey. These simple but impactful actions may have allowed RAs to bridge the many barriers that often affect HTR populations. It should be noted that the degree of trust generated by the humanizing component of follow-up may be influenced by the cultural competency of researchers [6,13,17]. The RAs contacting patients in this study were both female, spoke clear, fluent English, were both from Appalachian county’s within Geisinger’s primary coverage area, spoke with the region’s dialect, understood local colloquialisms, and spoke to all patients with an air of respect and an altruistic tone. Patients in HTR populations respond better to people with adequate competence of their culture or community [13,17,63]. These qualities have been described previously within models of cultural competence [6].

The success of this recruitment strategy and its associated supporting data and statistics are important to share with the scientific community given their potential use and broader application. We validated that including follow-up to email recruitment, especially in the form of human-mediated telephonic and email correspondence, significantly increased enrollment in web-based recruitment arms and can reach certain Appalachian, rural-dwelling, and older adult populations. This information is extremely valuable for researchers looking to find cost-effective ways for recruitment, while also targeting potential HTR populations and mitigating recruitment concerns related to COVID-19 and other health threats. Our study adds data to the literature that will provide more realistic comparisons for researchers looking to use email recruitment for large-scale recruitment studies, reach underserved or marginalized populations, and determine strategies to optimize web-based enrollment.

### Limitations

The overall population for this study were patients of European descent. The recruitment of an entirely European American sample was due to the sampling requirements for the overarching study [64], which will use population-based genomic analytic methods that have substantially improved power within homogeneous samples at this relatively small sample size. As a result, this study lacks racial and ethnic diversity and would need to be replicated in other types of HTR populations with more diversity to establish efficacy in other groups. In addition, the before and after follow-up data could be further refined to determine whether additional factors led to higher after call enrollment, such as the number of additional email links resent after the call. Patients could receive upward of 3 additional reminders after being contacted if they stated interest, requested a new email link, and partially completed the survey. These factors were not all built into REDCap and the emailing and calling process evolved as the needs of the project evolved. It should also be noted that we did not manipulate when the call occurred, so we cannot gauge whether patients would have still enrolled without a phone call if they had been given more time. As the proposed reasons for this study’s success are speculative, mixed methods studies including qualitative data will be needed to replicate and further support conclusions described here. This recruitment strategy would not work well in populations that had little or no access to telephone service or the internet. While our rural-dwelling, Appalachian, and older adult populations have reduced web-based access relative to more urban populations, a majority still have web-based access. In addition, currently we do not have access to whether each individual phone number is a mobile or landline, or information regarding service plans, or information on web-based access or plans, but future work should explore whether type of phone line impacts recruitment.

The COVID-19 pandemic also struck during the recruitment for this project. This recruitment arm of the project was able to continue recruitment, but recruitment percentages were reduced slightly (17% per batch before COVID-19 vs 15% per batch after COVID-19; see Table S2 in Multimedia Appendix 1). The effect of the pandemic on overall enrollment is difficult to determine. Web-based recruitment and increased time of this population at home may have given this project an advantage but the health and socioeconomic impact of COVID-19 on potential enrollees may have equally disadvantaged the recruitment process. These external factors, as well as a host of other external factors (season, weather, and holidays), make comparing individual batch success challenging.

### Conclusions

Recruitment in HTR populations has been notoriously difficult for researchers and these struggles are complicated by the need for cost-effective recruitment methods. Web-based recruitment of patients through a combination of email and follow-up with live person telephone calls and email correspondence is a safe, cost-effective method to attempt outreach to Appalachian, rural-dwelling, and older adult populations. The success of this web-based recruitment strategy may be due to the trust and validity established through a humanizing follow-up in the form of live person phone calls and email correspondence. Adding follow-up to email recruitment strategies positively impacts enrollment and can increase enrollment numbers in rural, Appalachian, and older adult populations using web-based recruitment methods.

The enrollment demographics and recruitment methods mentioned in this paper may help establish standards for recruitment success in various populations and study designs that attempt to reach Appalachian, rural-dwelling, and older adult populations. Reaching target populations and enrolling and retaining adequate sample sizes are imperative to the success of all studies. We further suggest that reporting participant demographics of enrollees and recruitment strategy statistics as a standard in future papers will fill knowledge gaps and assist future studies with recruitment goals.

## Appendix

Multimedia Appendix 1 Supplement with additional figure and tables depicting the geographical distribution of survey enrollment in Pennsylvania and detailed demographic breakdown of rural versus urban and Appalachian versus non-Appalachian for gender and age brackets.

## Abbreviations

DSM-5: Diagnostic and Statistical Manual of Mental Disorders (Fifth Edition)
HIPAA: Health Insurance Portability and Accountability Act
HTR: hard-to-reach
LGBTQIA+: lesbian, gay, bisexual, transgender, queer, intersex, and asexual
NIH: National Institutes of Health
PA: Pennsylvania
RA: research assistant
REDCap: Research Electronic Data Capture

## Data Availability

A deidentified summary file with patient enrollment counts can be made available upon reasonable request to the senior author.
