# Supplementary material for: Recruitment in Appalachian, Rural and Older Adult Populations in an Artificial Intelligence World: Study Using Human-Mediated Follow-Up
Source: JMIR Form Res. 2024 Aug 22;8:e38189. doi: 10.2196/38189 (PMC11377916; doi:10.2196/38189)
Supplement: Multimedia Appendix 1 [file formative_v8i1e38189_app1.docx]

**Multimedia Appendix 1.** Supplement with additional figure and tables depicting the geographical distribution of survey enrollment in Pennsylvania and detailed demographic breakdown of rural versus urban and Appalachian versus non-Appalachian for gender and age brackets.

Table of Contents:

1. *Figure S1A and S1B*: Pennsylvania Geographical Maps with recruitment densities in rural vs urban counties and in Appalachian vs non-Appalachian counties.
2. *Table S1*: Illustrates a breakdown of age brackets and gender in the enrollment rural and urban areas.
3. *Table S2:* Illustrates the recruitment differences during online recruitment pre-COVID19 and during COVID19.

**
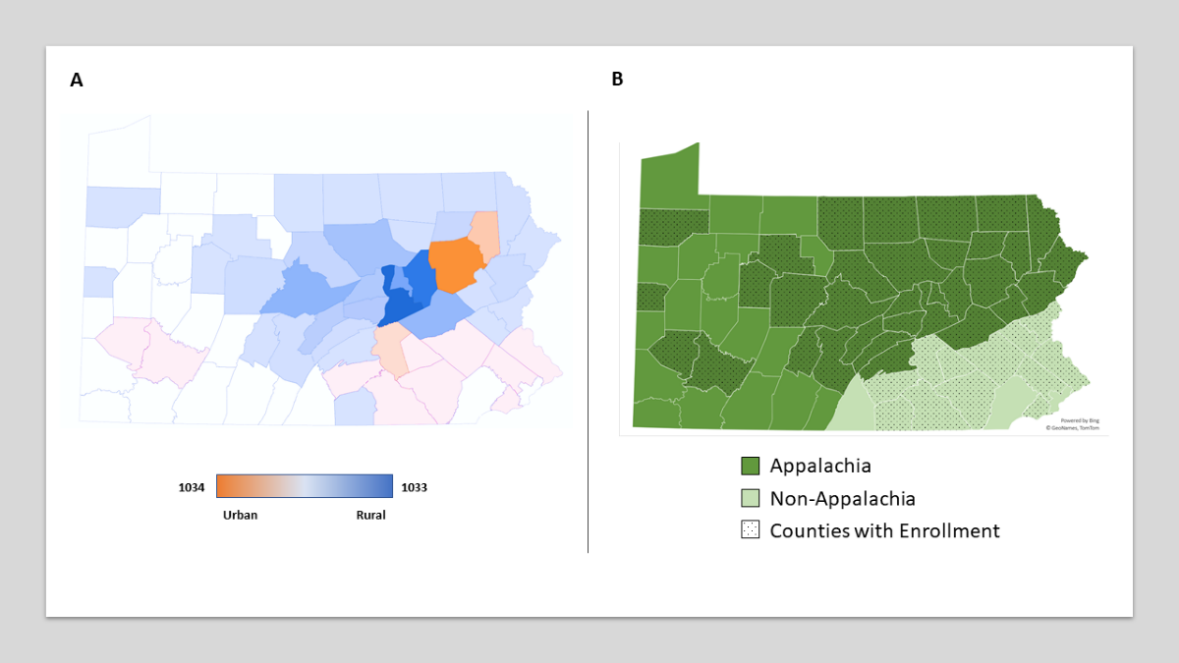
Figure S1A and Figure S1B:**

**Figure S1A is a geographical map of study enrollment in rural vs urban PA counties with blue indicating rural areas and orange indicating urban areas. Figure S1B is a geographical map that designates PA Appalachian and non-Appalachian counties. Dark green indicates Appalachian counties and light green indicates non-Appalachian counties. Counties with dots indicate counties with survey enrollment.**

**Table S1. Demographic breakdown of rural and urban populations in PA enrolled via email and follow-up recruitment arm of overall project.**

| **Population (n=6030)** | | | | | | | |
| --- | --- | --- | --- | --- | --- | --- | --- |
|  | | | | | | | |
|  | | **Female (N)** | **Female %** | **Male (N)** | **Male %** | **Grand Total (N)** | **Grand Total %** |
| **Rural** | | 3323 | 55.11% | 1278 | 21.19% | 4601 | 76.30% |
| Age (y) | 20-30  30-40  40-50  50-60  60-70  70-75 | 131  547  631  775  851  388 | 2.17%  9.07%  10.46%  12.85%  14.11%  6.43% | 17  104  164  294  422  277 | 0.28%  1.72%  2.72%  4.88%  7.00%  4.59% | 148  651  795  1069  1273  665 | 2.45%  10.80%  13.18%  17.73%  21.11%  11.03% |
| **Urban** | | 1048 | 17.38% | 381 | 6.32% | 1429 | 23.70% |
| Age (y) | 20-30  30-40  40-50  50-60  60-70  70-75 | 60  171  206  264  226  121 | 1.00%  2.84%  3.42%  4.38%  3.75%  2.01% | 6  28  55  85  130  77 | 0.10%  0.46%  0.91%  1.41%  2.16%  1.28% | 66  199  261  349  356  198 | 1.09%  3.30%  4.33%  5.79%  5.90%  3.28% |
| **Grand Total** | | 4371 | 72.49% | 1659 | 27.51% | 6030 | 100.00% |

**Table S2. Recruitment Pre-COVID19 vs during COVID19 for one year before COVID19 and one year during COVID19 during survey enrollment.**

| **Timeframe** | **Number of batches** | **Average % total enrolled per batch** | **Average % enrolled before follow-up** | **Average % enrolled after follow-up** |
| --- | --- | --- | --- | --- |
| **3/2019-2/2020** | 37 | 17% | 7% | 10% |
| **3/2020-2/2021** | 18 | 15% | 6% | 8% |
